# Unusual Cause of Artificial Urinary Sphincter Malfunction: Device Entanglement Resulting in Urethral Constriction and Urinary Retention

**DOI:** 10.7759/cureus.50440

**Published:** 2023-12-13

**Authors:** Mohamadhusni Zarli, Derek De Mann, Uzoma A Anele

**Affiliations:** 1 Urology, Nova Southeastern University Dr. Kiran C. Patel College of Osteopathic Medicine, Fort Lauderdale, USA; 2 Urology, University of Louisville School of Medicine, Louisville, USA; 3 Urology, University of Louisville School of Medicine, Louisville , USA

**Keywords:** stress urinary incontinence, malfunction, postoperative urinary retention, artificial urinary sphincter, surgical complication, malpositioned implant

## Abstract

Urinary retention following placement of an artificial urinary sphincter (AUS) is not an uncommon complication. We describe a unique case of urinary retention due to AUS entanglement causing urethral constriction in a 72-year-old male. He presented to the emergency department postoperatively following AUS placement with pelvic pain and incomplete emptying. Eventual cystourethroscopy demonstrated severe extrinsic urethral constriction despite the deactivation of the AUS device. Surgical exploration revealed an unexpected looping of the occlusive cuff, causing urethral constriction. Although the precise cause is not clearly known, we suspect that it may have been related to the process of connecting the tubing at the level of the abdomen. Regardless of the underlying etiology, this case highlights a unique complication and supports an assessment of the cuff with direct perineal inspection prior to wound closure and/or with cystourethroscopy.

## Introduction

The artificial urinary sphincter (AUS) is considered the gold standard treatment for moderate-to-severe urinary incontinence caused by sphincter insufficiency [1]. Since its debut in clinical practice in 1973, the AUS has undergone a series of modifications to improve long-term outcomes until the introduction of the AMS 800™ Artificial Urinary Sphincter (Boston Scientific Corporation, Marlborough, Massachusetts, United States) model in 1987 [2,3]. However, despite its introduction over four decades ago, the design remains unchanged, comprised of three core components: a cuff, a pressure-regulating balloon (PRB), and a control pump.

Demand for AUS devices is on the rise, driven by the escalating global incidence of prostatectomy surgery and consequent stress urinary incontinence (SUI) rates of up to 30% [4]. Despite good outcomes with AUS implantation, up to 25% of patients may require revision surgery, and this likelihood tends to increase over time. Potential complications that may prompt revision include urinary retention, infection, mechanical failure, urethral atrophy, and erosion [5]. We report a rare case of an unprecedented etiology of AUS malfunction resulting in acute urinary retention (AUR).

## Case presentation

Our case involves a 72-year-old male with a history of morbid obesity (BMI 44.6), stage 3B chronic kidney disease, and prostate cancer treated with radical retropubic prostatectomy and adjuvant radiation the following year. Later, he developed bilateral ureteral strictures which were subsequently managed with a robotic bilateral ureteroneocystostomy, and bothersome SUI requiring upwards of two to four diapers daily. Twelve years after prostatectomy, he underwent elective AUS placement following preoperative cystoscopic evaluation. Our standard approach involves a perineal and urethral dissection, measurement of the urethral circumference, development of the ectopic submuscular space, placement of the cuff with perineal closure, and insertion of the PRB and scrotal pump through the lower abdominal site.

During intraoperative assessment, the bulbar urethral circumference was measured to be 5 cm. Although the patient’s morbid obesity increased the technical difficulty of the procedure, the device placement was uncomplicated with ectopic right submuscular placement of a 61-70 cmH2O PRB. The device was cycled successfully multiple times at the conclusion of the operation. He was discharged on the same day postoperatively after voiding and confirmation of adequate emptying by ultrasound assessment of postvoid residual.

However, he presented to the clinic on postoperative day two with complaints of straining to void. Upon examination, the device pump was confirmed deactivated. A 12F urethral catheter was placed following an unsuccessful attempt to insert a 14 French catheter and 600 cc of urine was drained from the bladder. He returned to the clinic the following day where he successfully passed a trial of void; however, he presented to the emergency department (ED) again later that night due to difficulty urinating. His device was again noted to be deactivated, and he was discharged following spontaneous void and post-void residual volume (PVR) measurement of 1 cc.

**Figure 1:**
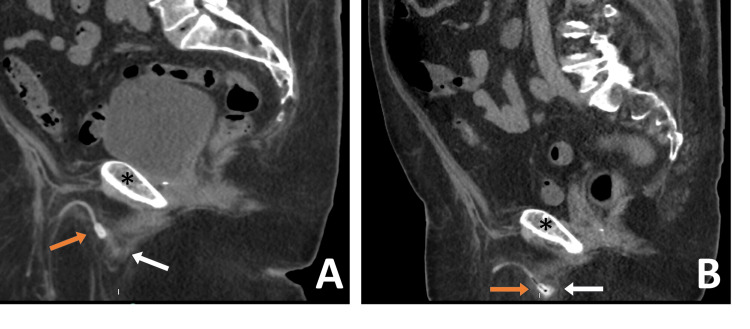
Sagittal CT scans. (A) Prior to surgical revision, the notch of the AUS tubing hiatus (orange arrow) can be seen to be misaligned with the urethra (white arrow) and at the level of the pubic bone (black asterisk); (B) Study performed for unrelated indication two months following surgical revision demonstrates the notch of the AUS tubing hiatus (orange arrow) is positioned in line with the urethra (white arrow) and inferior to the pubic bone (black asterisk). AUS: artificial urinary sphincter

On postoperative day 4, he returned to the ED with similar complaints and was found to have a PVR of 350 cc prompting 12F urethral Foley catheter placement. CT scan of the abdomen and pelvis demonstrated a suspicious abnormality of the urethral cuff (Figure 1). He was admitted to the hospital and a bedside cystoscopy was subsequently performed which revealed a severe concentric constriction within the bulbar urethra at the cuff site despite the device being in the deactivated state.

**Figure 2:**
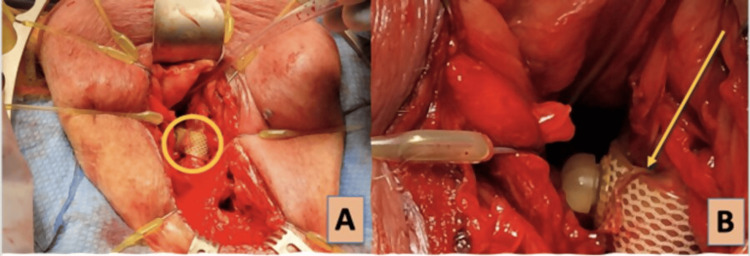
(A) A deep perineal dissection carried down to the urethra. The bulbospongiosus muscle is retracted laterally with yellow hooks and AUS device is identified (circle); (B) Magnified view of AUS device with the cuff’s body advanced within the fastener loop (arrow) AUS: artificial urinary sphincter

On postoperative day 7, the patient was taken to the operating room for flexible cystoscopy and perineal exploration. Cystoscopy once again demonstrated the constriction of the bulbar urethra at the AUS cuff site. The cuff was then cycled under direct vision without change in the constriction. The perineal incision was reopened and explored to the level of the bulbar urethra where the cuff was identified. The cuff was found to be entangled in itself as the notch of the tubing origin passed beyond the fastener loop resulting in an over-tightened cuff and severe urethral constriction (Figure 2). The cuff was then released from the loop and then adjusted properly. A repeat cystoscopy showed restoration of the urethral lumen caliber along with some superficial mucosal bruising. Notably, there was no evidence of cuff erosion or damage. The AUS was found to be functioning appropriately, obviating the need for device replacement. His device was eventually activated over seven weeks after surgery without difficulty or issue. Nearly three months following surgery, he reported successful functionality of his device and lack of any further SUI to the point of no longer requiring the use of incontinence products.

## Discussion

The AMS-800 Artificial Urinary Sphincter has excellent outcomes regarding urinary continence and offers remarkable improvement in quality of life for patients with SUI [5]. However, AUS placement is not without potential complications which include urinary retention (31%), urethral atrophy (9.6-11.4%), urethral erosion (3.8-10%), device-related infection (6%), and skin infection (1%) [6].

The first main component of the AUS is the 2 cm wide occlusive cuff, which is typically placed encircling the bulbar urethra and can have various sizes ranging from 3.5 cm to 11 cm determined by the urethral circumference. The second main component is the PRB, which regulates pressure to the cuff and ranges from 51 cmH2O to 80 cmH2O. It can be placed in different locations including the space of Retzius, a submuscular position, or via a counter incision in a preperitoneal space [5]. Urologists may prefer the submuscular positioning of the PRB, which offers comparable functional outcomes to the traditional positioning in the space of Retzius while minimizing associated complications in re-operative cases if required subsequently [7,8]. The final component is the control pump, which consists of two parts, an upper unit and a lower unit, and is typically placed in the scrotum within a subdartos pouch. The upper unit contains the deactivation button and resistor valves, while the lower unit contains a bulb that is squeezed by the patient to transfer fluid from the compressive cuff to the PRB, allowing for micturition [5].

A comprehensive cohort study conducted by Linder et al. highlighted postoperative urinary retention as the most common complication following AUS placement, occurring in 31% of cases. This urinary retention can often be attributed to multiple factors, most commonly urethral edema resulting from periurethral dissection, but also from errors in cuff sizing, or surgical injuries that may go unnoticed [9].

In the current report, a unique and unreported complication was encountered. The occlusive cuff inexplicably tightened around itself through the fastener loop, resulting in severe urethral constriction and subsequent urinary retention. This unprecedented occurrence presented a significant challenge, as no prior literature had addressed such an etiology for AUS malfunction. The exact cause of this phenomenon remains elusive; however, we hypothesize that factors such as surgical technique, anatomical variations, and/or cuff material properties may have played a role. The cuff is typically placed around the circumference of the urethra and the loop is secured in place around the notch of the tubing hiatus. The protruding tab is then cut after this positioning is confirmed. We suspect that it is possible that there was excessive manipulation of the cuff tubing through the abdominal incision during the tubing connection process which may have forced the body of the cuff through the loop. Because these connections were made after the perineum was closed, the cuff could not be directly assessed.

Despite the rarity of this complication, we have adjusted our practice to delay the closure of the perineum until after the connections are made to assess the cuff. This case underscores the utility of cuff evaluation either through cystourethroscopy or direct assessment via the perineal incision prior to closure at the time of initial device insertion. In this case, the resolution required re-exploration of the perineum to carefully examine and release the cuff, ultimately restoring proper device function and relieving the urinary retention.

## Conclusions

This case introduces a rare and previously unreported complication in an AUS recipient. Despite the established effectiveness of AUS in treating SUI, this incident revealed a novel etiology of AUS malfunction leading to AUR. In this unique case, the occlusive cuff inexplicably became entangled, causing severe urethral constriction and urinary retention, an occurrence not previously documented. Although the exact cause remains unclear, perineal exploration was required to determine and ultimately restore proper device function. This case emphasizes the utility of intraoperative cuff evaluation at the time of device implantation.
